# Three mitochondrial genomes of early-winged insects (Ephemeroptera: Baetidae and Leptophlebiidae)

**DOI:** 10.1080/23802359.2021.1974966

**Published:** 2021-09-17

**Authors:** Sereina Rutschmann, Ping Chen, Changfa Zhou, Michael T. Monaghan

**Affiliations:** a Leibniz Institute of Freshwater Ecology and Inland Fisheries (IGB), Berlin, Germany; b Berlin Center for Genomics in Biodiversity Research, Berlin, Germany; c University of Potsdam, Institute of Biochemistry and Biology, Potsdam, Germany; d College of Life Sciences, The Key Laboratory of Jiangsu Biodiversity and Biotechnology, Nanjing Normal University, Nanjing, China

**Keywords:** *Baetis*, *Cloeon*, *Habrophlebiodes*, mayfly, mitochondrial phylogeny

## Abstract

Mayflies (Ephemeroptera) are a semi-aquatic insect order with comparatively few genomic data available despite their phylogenetic position at the root of the winged-insects and possession of ancestral traits. Here, we provide three mitochondrial genomes (mtgenomes) from representatives of the two most species-rich families, *Baetis rutilocylindratus* and *Cloeon dipterum* (Baetidae), and *Habrophlebiodes zijinensis* (Leptophlebiidae). All mtgenomes had a complete set of 13 protein-coding genes and a conserved orientation except for two inverted tRNAs in *H. zijinensis.* Phylogenetic reconstructions using 21 mayfly mtgenomes and representatives of seven additional orders recovered both Baetidae and Leptophlebiidae as well supported monophyletic clades, with Ephemeroptera as the sister-taxon to all other winged insects (i.e. Odonata and Neoptera).

The number of published molecular markers and genomes of the semi-aquatic insect order Ephemeroptera (mayflies) is low compared to the evolutionary diversity that the order represents (Almudi et al. 2020; Hotaling et al. 2020; Xu et al. 2020). The mayflies comprise >3000 species in around 42 families and >400 genera, and fossil records date back to the lower Carboniferous (Barber-James et al. 2008; Ogden et al. 2009). The monophyly of the order is well established (Hovmöller et al. 2002; Ogden et al. 2009), although its relationship to Odonata and all other winged insects remains a long-standing controversy (Hovmöller et al. 2002; Ogden and Whiting 2003; Misof et al. 2014). Within the mayflies, the relationships of several major clades remain unresolved (e.g. Miller et al. 2018; Xu et al. 2020). The availability of additional molecular data will help to shed light on these unresolved issues.

Here, we newly sequenced three mitochondrial genomes (mtgenomes) of the two most diverse Ephemeroptera families: *Baetis rutilocylindratus* Wang, Qin, Chen & Zhou, 2011 and *Cloeon dipterum* L. 1761 from the Baetidae, and *Habrophlebiodes zijinensis* Gui, Zhang & Wu, 1996 from the Leptophlebiidae. Specimens of *H. zijinensis* and *B. rutilocylindratus* were collected in Zijin Hill, Nanjing, China (32.055 latitude, 118.859 longitude). Their DNA was extracted using the DNeasy^®^ Blood & Tissue (Qiagen, Hilden, Germany) kit. Initially, four fragments of the mtgenome were amplified via standard PCR using universal primers (Simon et al. 1994) and subsequently the sequences were extended by newly designed primers (see Supplementary Information Table S1). All purified amplification products were sequenced successively in both directions using a 3130 xl (Applied Biosystems, Waltham, MA) capillary sequencer. The mtgenome of *C. dipterum* was obtained from DNA of laboratory-reared subimago specimens (full siblings). The laboratory-founder individual was collected in the United States of America (39.865 latitude, −75.818 longitude). Analysis of nuclear and mitochondrial markers established the species as one of six putative species of *C. dipterum* s.l. with a geographical distribution in North America and Europe (Rutschmann et al. 2017). Specimens and DNA of the species are stored at The Key Laboratory of Jiangsu Biodiversity and Biotechnology (Nanjing, China, voucher numbers NNU-20110910, NNU-2010710) and the Museum of Zoology (Lausanne, Switzerland, voucher number US). We extracted the DNA, prepared a shotgun library, and sequenced four lanes on a 454 GS FLX pyrosequencer (Roche Diagnostics, Rotkreuz, Switzerland). Sequence reads were trimmed and *de novo* assembled as described in Rutschmann et al. (2017). In order to extract the mtgenome, we performed BLASTN searches (Altschul 1997) using all assembled contigs to query the NCBI database. We mapped all matching reads back to the mtgenome with BWA (Li and Durbin 2009), using the BWA-SW algorithm (Li and Durbin 2010). All mtgenomes were annotated using MITOS (Bernt et al. 2013) and tRNAscan-SE v.1.21 (Lowe and Eddy 1997).

The sequences are deposited in GenBank with the accession numbers GU936204, MW149047, and GU936203. They were 14,883 bp (*B. rutilocylindratus)*, 15,407 bp (*C. dipterum*), and 14,355 bp (*H. zijinensis*) long, whereby the one of *B. rutilocylindratus* was complete (i.e. circular). All three contained the entire set of 13 protein-coding genes (PCGs), two rRNAs, and 22 (*B. rutilocylindratus* and *C. dipterum*) or 19 tRNAs (*H. zijinensis*). Gene order was conserved with the exception of two inverted tRNAs (arginine and alanine) in *H. zijinensis*.

Bayesian phylogenetic trees were reconstructed based on the nucleotide dataset of the 13 PCGs using MrBayes v.3.2.7 (Ronquist et al. 2012). We aligned each PCG separately using MAFFT v.7.407 (L-INS-I algorithm with default settings, Katoh and Standley 2013) and inferred the tree based on the concatenated alignment, applying the best-fit evolutionary models and partitioning-schemes as inferred with PartitionFinder v.2 (https://github.com/brettc/partitionfinder). *Petrobius brevistylis* (Archaeognatha, NC_007688) was specified as outgroup. The recovered phylogeny (Figure 1) was highly supported, including all nodes with Bayesian posterior probability (BPP ≥ 0.95). The Ephemeroptera were clustered as sister group to the remaining winged-insects, comprising the Odonata and Neoptera. Within mayflies, the Leptophlebiidae were recovered as sister group to all other representatives. The Caenidae, Teloganodidae, and Baetidae formed a highly supported monophyletic clade.

**Figure 1.**
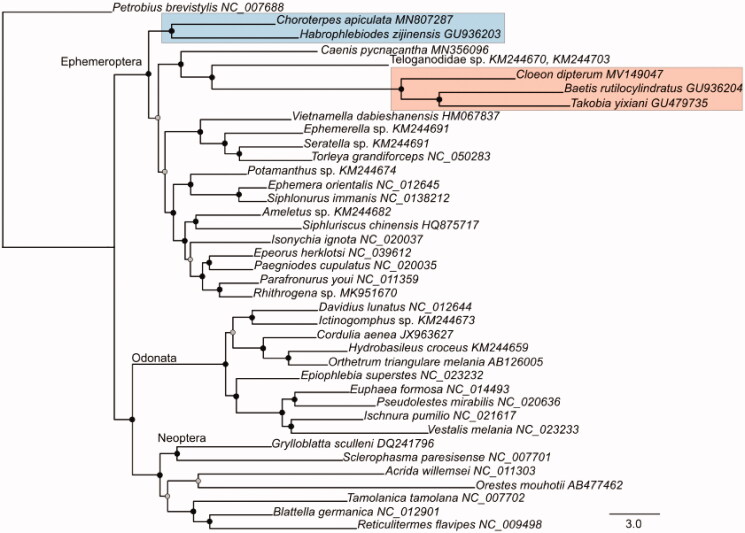
Phylogenetic relationships of insect major orders using Bayesian inference reconstruction based on the nucleotide dataset of the 13 mitochondrial protein-coding genes. Filled circles indicate supported nodes; whereby black circles represent Bayesian posterior probability (BPP) ≥0.99, and shaded circles BPP ≥0.95. The two families of Baetidae and Leptophlebiidae are highlighted. The GenBank accession numbers of all specimens are provided in the tip labels.

## Supplementary Material

Supplemental Material

## Data Availability

The data that were newly sequenced as part of this study are available in NCBI (https://www.ncbi.nlm.nih.gov/, accession numbers GU936204, MW149047, and GU936203). The data are associated with BioSample SAMN03202660 and BioProject PRJNA268073, and the raw sequencing reads are available from the Sequence Read Archive SRP050093.
